# Optimized DOX Drug Deliveries via Chitosan-Mediated Nanoparticles and Stimuli Responses in Cancer Chemotherapy: A Review

**DOI:** 10.3390/molecules29010031

**Published:** 2023-12-20

**Authors:** HafizMuhammad Imran, Yixin Tang, Siyuan Wang, Xiuzhang Yan, Chang Liu, Lei Guo, Erlei Wang, Caina Xu

**Affiliations:** 1 Department of Biochemistry, College of Basic Medical Sciences, Jilin University, Changchun 130021, China; imran22@mails.jlu.edu.cn (H.I.); tangyx21@mails.jlu.edu.cn (Y.T.); wsy15981810884@163.com (S.W.); jlbsyxz123@163.com (X.Y.); drliuchang@jlu.edu.cn (C.L.); guolei981112@163.com (L.G.); 2 College of Food Science and Engineering, Jilin University, Changchun 130062, China

**Keywords:** chitosan NPs, drug delivery, drug resistance, stimuli-sensitive, chemotherapy

## Abstract

Chitosan nanoparticles (NPs) serve as useful multidrug delivery carriers in cancer chemotherapy. Chitosan has considerable potential in drug delivery systems (DDSs) for targeting tumor cells. Doxorubicin (DOX) has limited application due to its resistance and lack of specificity. Chitosan NPs have been used for DOX delivery because of their biocompatibility, biodegradability, drug encapsulation efficiency, and target specificity. In this review, various types of chitosan derivatives are discussed in DDSs to enhance the effectiveness of cancer treatments. Modified chitosan–DOX NP drug deliveries with other compounds also increase the penetration and efficiency of DOX against tumor cells. We also highlight the endogenous stimuli (pH, redox, enzyme) and exogenous stimuli (light, magnetic, ultrasound), and their positive effect on DOX drug delivery via chitosan NPs. Our study sheds light on the importance of chitosan NPs for DOX drug delivery in cancer treatment and may inspire the development of more effective approaches for cancer chemotherapy.

## 1. Introduction

Chemotherapy has played an important role against cancer cells. However, cancer ranks as the top cause of death globally [1]. Mostly, chemotherapeutic drugs are unable to penetrate cancer cells due to intracellular efflux and the expression of p-glycoprotein [2]. However, numerous critical mechanisms are responsible for interactions between tumors and chemotherapeutic drugs. Correspondingly, alterations in protein structures due to genetic mutation could resist drugs binding with proteins [2]. Sometimes, drugs are unable to kill the cancer cells due to drug resistance during cancer genesis. Conventional chemotherapy has shown some severe side effects; for example, myeloid leukemia due to alkylating agents [3]. Many therapeutic agents and drug combinations with effective toxicity against tumor cells have been developed for chemotherapy. The main task in cancer treatment is to transport optimal quantities of drugs to the tumor positions without damaging normal cells [4].

Innovation in pharmaceutical nanotechnology has provided convenience in the field of medicine, particularly in the drug delivery area. Drug delivery through nanostructures is a great method for delivering therapeutic agents to the target site in a controlled environment for a desired period [5,6]. A large number of polymers have been developed for the controlled and targeted delivery of drugs to specific sites. These polymeric nanocomposites have exhibited numerous advantages in the field of biomedical applications [7,8]. Physiochemical properties, the surface potential, the size of nanocomposites, and their impact on cells are the main factors to consider when choosing effective delivery agents. These nanocomposites boost targeted drug absorption and protect against premature drug interactions with the healthy cells. These nanocarriers not only block premature drug interactions but also regulate the drug distribution profile and pharmacokinetics [9]. Researchers are directing their attention toward the characteristics and combinations of biopolymers with drugs in order to improve drug delivery for cancer treatment [10]. They have developed different therapeutic drugs and drug carriers for cancer treatment using nanotechnologies, such as NPs, nanocapsules, liposomes, dendrimers, nanocrystals, emulsions, and micelles [11]. NPs can deliver the drug to specific target sites, improve the solubility, and enhance the drug circulation and drug concentration around the cancer cells through the permeability and retention effect [12,13,14]. Chitosan NPs have gained substantial attention due to their outstanding biocompatibility, low toxicity, biodegradability, stimulus sensitivity, and cost-effectiveness [15].

Chitosan is a popular biopolymer because it contains active amino groups that allow for the attachment of numerous functional groups under mild reaction conditions. Furthermore, these amino groups are responsible for the cationic natures of chitosan as well as its water-soluble feature at low pHs [16]. Furthermore, chitosan’s major amine functional groups are involved in mucoadhesion, regulated drug administration, in situ gelation, permeability induction, efflux pump inhibition, and colon targeting [17]. Chitosan is a biomaterial that has antibacterial action, minimal immunogenicity, great biocompatibility, and biodegradability. The biodegradability of chitosan also plays an important role in DDS; chitosan can be degraded by chemical processes and enzyme catalysis. Enzyme catalysis depends on the degree of acetylation as well as accessibility of amino groups [16,18,19]. Chitosan nanocomposites, mostly with typical particle sizes smaller than 100 nm, are useful for biological applications because they are nontoxic, cost-effective, and sustainable [20]. Furthermore, because of their thermal and mechanical stability, chitosan nanocomposites may be produced in a variety of physical forms, such as films, powder, fiber, meshes, beads, membranes, porous frames, and hydrogels [21].

Because of its extensive formulation possibilities, chitosan is widely used in DDS. Furthermore, chitosan has important features, such as its hemostatic, anticarcinogenic, anticholestermic, fungistatic, and bacteriostatic properties [22,23]. Chitosan is used in a variety of forms, depending on the desired functionalities and manufacturing techniques, such as NPs, microspheres, capsules, hydrogels, conjugates, and more. Certain features of chitosan-mediated drug delivery systems, such as particle size, toxicity, thermal and chemical stability, and release kinetics, are largely dependent on the preparation procedures used [24]. Because chitosan NPs can be designed to target specific tissues, the cell-specific targeting of chitosan NPs is a promising approach for minimizing nonspecific interactions, increasing the local drug concentration, and lowering the toxicity and side effects associated with systemic administration [25]. Modifying NPs with peptides, antibodies, aptamers, or small molecules allows for targeted distribution. These targeting strategies not only enable the use of reduced therapeutic doses but also facilitate drug delivery to receptors present on cancer cells [26].

In the past, several therapeutic drugs have been used to treat cancer patients, among which doxorubicin (DOX) is a very effective drug against cancer cells [27]. DOX is capable of controlling various cancers, like gastric cancer, breast cancer, and bladder cancer [28,29]. However, clinical applications of DOX are limited due to toxicity in the cardiac, gastrointestinal part, and normal cells [30]. Moreover, the integration of DOX into the nucleus and cytoplasm is crucial to kill cancer cells [31,32]. Many studies have indicated the ability of nanocarriers to control DOX resistance and increase DOX accumulation to eliminate tumor cells. The codelivery of DOX with other anticancer agents also enhances the DOX capability and reduces the DOX resistance [33]. It has also been noted that the codelivery of DOX and tumor-suppressor genes increases the anticancer activity of DOX. Some internal and external stimulus responses also affect the anticancer activity of DOX. Using a biocompatible DDS is essential to cure cancer cells and enhance the therapeutic properties of DOX [34]. Nano-DDSs are developed for DOX delivery to reduce the cytotoxic effect through alteration in the rate of drug delivery and tissue distribution. For efficient DOX delivery, NPs are a good option to cross barriers and induce the DOX accumulation in the tumor microenvironment. NPs protect DOX against hydrolysis inactivation and enhance solubilization into cancer cells. These NPs also increase the retention time of DOX in tumor cells, which is beneficial in cancer treatment. For these purposes, chitosan NPs gained much attention. Many studies have been conducted on chitosan NPs, demonstrating their efficiency in drug conjugation and low toxicity to healthy cells [35]. These findings highlight the prominence of chitosan-based NPs for delivering DOX effectively.

Stimulus-sensitive drug delivery has gained consideration due to differences in the environment surrounding cancer cells [36,37]. Endogenous stimuli consist of redox, enzymes, pH, and temperature [38,39]. Exogenous stimuli consist of light, magnetic fields, and ultrasound [40,41]. Modified-chitosan-based NPs are considered one of the best drug delivery carriers among a variety of biomaterial nanocomposites due to their specific functional groups (amino and acetamido). This review describes the importance of chitosan NPs, as well as their properties, modifications, and the preparation for DDS in cancer chemotherapy. The mechanism of action of DOX and its delivery via chitosan NPs, along with different combinations, for the effective treatment against cancerous cells are explained. Various stimulus responses have also been discussed for DOX delivery to the tumor cells by using chitosan NPs. This study contributes to the comprehension of chitosan NPs in delivering DOX for enhanced chemotherapeutic strategies against cancer.

## 2. Biological Importance of Chitosan

**Figure 1:**
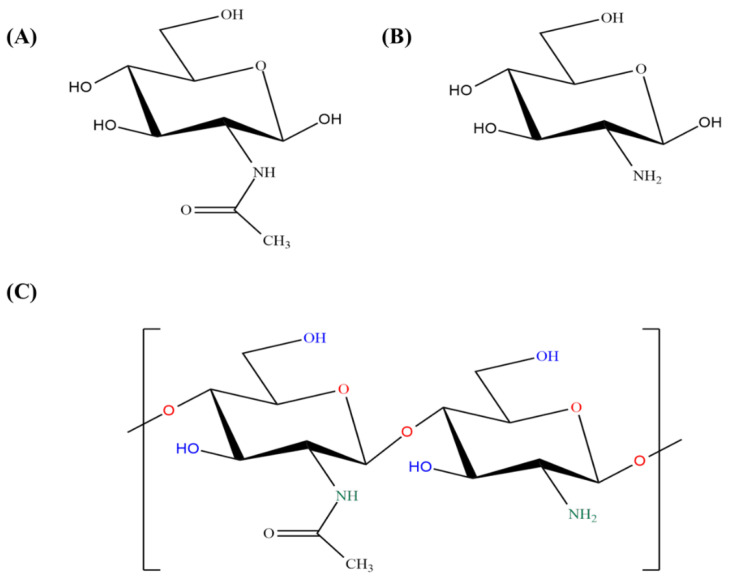
(**A**) N-acetyl-D-glucosamine unit, (**B**) glucosamine unit, and (**C**) β 1-4 linkage in the structure of chitosan.

Chitosan is a naturally occurring polysaccharide that has been used in wound healing [42], drug deliveries of chemotherapeutic agents [43,44], and the delivery of genes [45]. It is the second most abundant biopolymer and can be obtained through the hydrolysis of the N-acetyl glucosamine unit of chitin in alkaline conditions [46]. Chitosan is composed of deacetylated units, like glucosamine sugar, and acetylated units, like N-acetyl-D-glucosamine sugar, linked via the β 1 to 4 linkage (Figure 1). There are three main types of chitins, referred to as α-chitin, β-chitin, and γ-chitin, based on the arrangement of the polymer chains [46]: α-chitin, which showed antiparallel arrangements of polymer chains; β-chitin, which showed parallel arrangements of polymer chains; γ-chitin, which showed irregular arrangements of polymer chains. Generally, a pH fluctuation can degrade the chitosan NPs, while different modifications have increased the stability of chitosan [47]. The biodegradability of chitosan can be increased through the physical ionic gelation method by using polyanionic materials, such as tripolyphosphate (TPP) [48]. The molecular weight and the degree of deacetylation (DDA) are responsible for the physiochemical properties of chitosan, including the solubility and viscosity [49]. Chitosan biodegradability is inversely proportional to the molecular weight and DDA [50]. The capability of drug loading and releasing is directly dependent on the molecular weight of chitosan, and a high molecular weight results in a slow release compared to low-molecular chitosan [49]. Chitosan with high deacetylation exhibits optimum functioning and toxicity. The term “zeta potential” refers to the electrokinetic potential of charged particles in a fluid, and it is commonly measured to understand the nature and stability of particles in various environments, including living systems. Due to the protonation of the amino group in an acidic environment, chitosan acquires a positive charge [51]. Moreover, the ionic strength and pH of the solution are parameters responsible for the value of the zeta potential [52]. Surface chemistry and a high surface charge (positive or negative) are the two main factors for the delivery of drug-loaded particles. Previous studies have elaborated that the electrostatic forces between the negatively charged cancer cell membranes and the positively charged chitosan extend the retention time of chitosan at the target site and enhance the absorption capacity [53]. The positive charge of chitosan is also beneficial for cross-epithelial drug delivery, increasing paracellular permeability [54]. Moreover, the positive charge of chitosan can prevent the degradation of the therapeutic agent from passing through the lysosome via the proton sponge effect [55].

### 2.1. Clinical Medicinal Use

Chitosan and its derivatives are used in the therapeutic treatment of a variety of disorders, showing promising benefits in cholesterol reduction, immunomodulation, hemostasis, and the management of diseases such as cancer and diabetes [56]. Furthermore, they are suitable in avoiding oral diseases, such as periodontitis, mouth ulcers, and dental caries [57]. Furthermore, in DDS, these compounds act as outstanding release-controlling agents. Chitosan NPs behave as drug carriers in a variety of forms, including beads, capsules, bioadhesive gels, and films, and can be administered by oral, parenteral, transdermal, ocular, and other routes [58]. Chitosan-based NPs are of great interest to researchers due to their simplicity, stability, efficiency, and capability for targeted delivery.

### 2.2. Biomaterials

Chitosan and its derivatives can be used to produce a variety of medicinal biomaterials, including surgical sutures, medical membranes, and tissue-engineering scaffolds [59]. Notably, it is completely safe because it may be absorbed by the human body without the need for postoperative intervention. Chitosan is useful in tissue engineering for mending skin, cartilage, bone, liver tissue, and injured nerves [60]. For tissue engineering, several chitosan-based materials, such as porous scaffolds and gels, have been investigated [61]. Chitosan-based scaffolds, which act as temporary 3D frameworks, promote cell growth and guidance, resulting in the formation of desired tissues. The chitosan-based scaffolds disintegrate or merge with the tissue during culture. Chitosan has been shown to increase cell proliferation while reducing extralocal irritation. Furthermore, chitosan-based scaffolds may be used for gap filling as well as the regulated release of bioactive compounds (growth factors, nutrients).

### 2.3. Drug Delivery

Significant chitosan-based delivery systems for the mucosal delivery of peptides, proteins, polar medications, immunizations, and DNA have been described on a regular basis. So far, no major inflammatory or allergic responses have been reported in association with the implantation, injection, topical application, or ingestion of chitosan-based biomaterials in the human body [62]. Chitosan has the potential to be a drug control–release carrier. Several chitosan formulations (solutions, suspensions, gels, microemulsions, and powders) with mucoadhesive and penetration-enhancing capabilities have been proposed for the nose-to-brain delivery of drugs. Researchers have been encouraged to construct alternative nucleic acid delivery vectors due to the poor transfection efficacy of naked nucleic acids delivered in vitro and in vivo [63,64]. A chitosan-based delivery method (indirect method) was successful for nonviral gene therapy [65]. Chitosan and its derivatives have the ability to bind nucleic acids via electrostatic interactions and can be endocytosed into cells without dissolving the chitosan–DNA complex. Improved membrane adherence and the lysosomal escape of encapsulated DNA allow for efficient cell transfection [66].

## 3. Chemical Modification of Chitosan for Drug Delivery

The electrostatic interactions, molecular weight, and DDA affect the biological role of chitosan [67,68]. Numerous studies have focused on chemically altering the active groups of chitosan to enhance its application in immunotherapy. Various chemical approaches used for chitosan modification include N, O-substitution reactions, carboxymethylation, acylation, thiolation reactions, alkylation, quaternization, phosphorylation, sulfation, grafting, etc. For example, compared to chitosan, trimethyl chitosan (TMC) is easily soluble in alkaline and neutral solutions due to the presence of the protonated group (-N+(CH_3_)_3_) [69]. Such a positive charge favors drug delivery and enhances the mucoadhesion and diffusion capacity. As an immune potentiator, quaternized chitosan can encourage the production of proinflammatory cytokines through the activation of innate immune Toll-like receptors (TLRs) and also behaves as a delivery carrier to transport drugs and target antigen-presenting cells (APCs) [70]. The immune capability of quaternized chitosan is influenced by both the molecular weight and the degree of quaternization (DQ) [71]. Quaternized chitosan with a moderate DQ has an improved immunomodulatory effect, resulting in better outcomes. For example, quaternary ammonium derivatives of chitosan, such as N-(2-hydroxyl) propyl-3-trimethyl ammonium chitosan chlorides, are nontoxic, water-soluble, stable, and positively charged chitosan nanocomplexes in the physiological environment [72,73]. These nanocomposites, owing to their unique aspects, such as good permeability and mucoadhesion, have been considered potent nanocarriers for the delivery of therapeutic agents to cancer tissues [73]. Carboxymethyl chitosan (CMC) is synthesized by presenting carboxymethyl groups to the chitosan. It is an amphiphilic material due to the existence of carboxyl groups in its chemical structure [74]. CMC shows a pH dependency in water solubility, as it is insoluble at a pH from 3.5 to 6.5, but fully soluble except in this range [75]. CMC controls tumor growth and increases immunity in the living body by increasing the serum levels of IL-2 and tumor necrosis factor-α (TNF-α) [76]. Using CMC as a carrier with a vaccine can improve the uptake of dendritic cells, which is beneficial for the activation of the downstream immune system [77]. In addition, thiolated chitosan (TC) is obtained through the covalent linkage of thiol groups to chitosan [78,79]. TC exhibits adherence and good permeability, playing the role of a carrier by creating a mercaptan bond through transmembrane proteins that are efficiently internalized via cell endocytosis [80,81]. The thiol group empowers thiolated chitosan molecules to cross-link with each other, aiding in in situ gelation and providing mechanical stability to the carrier, thereby achieving effective drug release [82,83]. Glycated chitosan (GC) is a water-soluble complex produced by the reaction of galacturonic groups with chitosan [84]. GC has been used in phototherapy with TNF-α to treat tumors [85]. Several modified approaches of chitosan have been used for different objectives, which are valuable in immunotherapy, as shown in Table 1.

## 4. Methods for Chitosan NP Preparation for Drug Delivery

### 4.1. Emulsion Cross-Linking Method

The cross-linking method is commonly used for the synthesis of chitosan NPs. In this method, a water-in-oil emulsion is prepared by adding a chitosan solution in an oil phase. A suitable surfactant, such as span 80, is added to improve the stability of the aqueous droplets. After that, glutaraldehyde is mixed dropwise to cross-link with the NH_2_ groups of chitosan [98]. Then, the solution is filtered to obtain NPs, washed with alcohol, and dried [99]. In the cross-linking method, the concentration of chitosan and glutaraldehyde directly influences the physicochemical properties of the chitosan NPs, including the drug release, size, degradation rate, and zeta potential. For example, a higher concentration of chitosan with respect to glutaraldehyde may result in a denser network, thereby affecting the drug release from the chitosan NPs. Similarly, a different concentration of glutaraldehyde can lead to different degrees of cross-linking, influencing the size of the chitosan NPs. Chitosan-grafted polymers have been prepared by using a glutaraldehyde cross-linker with the carboxymethyl chitosan micelle surface [100]. In this method, stearic acid-grafted chitosan oligosaccharides (CSO-SAs) have been prepared for drug delivery. A chitosan solution was synthesized by adding hydrochloric acid and raising the temperature to 50 °C. After that, the chitosanase enzyme was added and the solution was filtered. CSO was mixed with the steric acid and subjected to sonication treatment to obtain the required product. After that, the drugs were introduced. Amphiphilic CSO-SA self-aggregated to produce nanosized materials. Different degrees of amino groups can change both the rate at which drugs are delivered and the solubility of the micelles. Similarly, glutaraldehyde cross-linking with the surface of NPs can enhance the capacity to control drug release [18].

### 4.2. Precipitation or Coacervation Method

**Figure 2:**
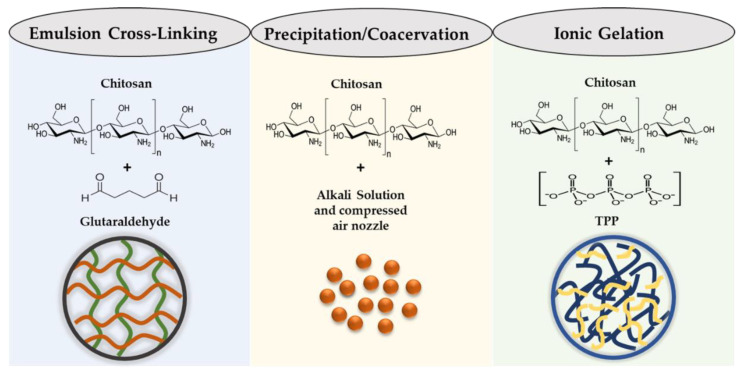
Several approaches (the emulsion cross-linking method, precipitation/coacervation method, and ionic gelation method) of chitosan NP preparation [18].

Chitosan is insoluble in an alkaline pH. This physicochemical property of chitosan is utilized in this method. First, an acidic solution of chitosan (usually acetic acid) is prepared. This chitosan solution is propelled by using a compressed nozzle or spray technique into an alkaline environment solution, such as ethane diamine, NaOH–methanol, and sodium hydroxide, to prepare coacervate droplets. After that, the droplets are filtered and washed with distilled water. Mao and his colleagues synthesized chitosan–DNA NPs using a coacervation approach under optimum conditions. They synthesized chitosan–DNA NPs with the addition of chloroquine. Initially, the chitosan and DNA mixtures were heated at 50–55 °C independently and mixed quickly. Chloroquine was added and washed to obtain the desired product. According to this method, the DNA reacted with chitosan via electrostatic forces, and then a separation phase was performed to prepare the coacervates. Sodium sulfate was added to facilitate phase separation. The pKa value of the amino groups of chitosan was 6.5, which means that most amine groups were protonated at a pH of 5.5. In this way, the pH-stable NPs can be prepared without the need of cross-linking methods. Figure 2 illustrates the different preparation methods of the chitosan NPs under various environmental conditions to achieve the desired final product with different combinations.

### 4.3. Ionic Gelation Method

The ionic gelation method has gained substantial attention. According to this approach, chitosan NPs undergo reversible physical cross-linking with oppositely charged TPP through an electrostatic connection. To protonate the amine groups of chitosan, it was mixed with the aqueous acidic solution (acetic acid). Subsequently, this solution was added dropwise to the TPP solution with continuous stirring [71,101]. The surface of the chitosan NPs has amine groups that can be modified through various ligands. Wen Fan et al. prepared chitosan/TPP NPs using an ionic gelation approach. Initially, low-molecular-weight (LMW) chitosan was dissolved in acetic acid, with the acetic acid concentration being 0.4 times that of the chitosan. Afterward, the chitosan solution was mixed overnight on a magnetic stirrer and adjusted to a pH of 4.7–4.8 by adding sodium hydroxide solution. Subsequently, the entire solution was treated with a syringe filter to remove the insoluble particles. Additionally, TPP was mixed with distilled water and passed through a syringe filter. Next, the chitosan solution was heated to 60 °C and placed on a magnetic stirrer. This magnetic stirrer was then placed in a chest freezer at 2–4 °C and the TPP solution was added rapidly. The concentration of both TPP and chitosan can alter the particle size. Various parameters, such as the pH, concentration, and stirring speed, were carefully adjusted to synthesize the NPs with an enhanced drug-loading capability, which is beneficial for biomedical applications, such as the delivery of genes [102].

## 5. DOX Structure and Mechanism of Action

**Figure 3:**
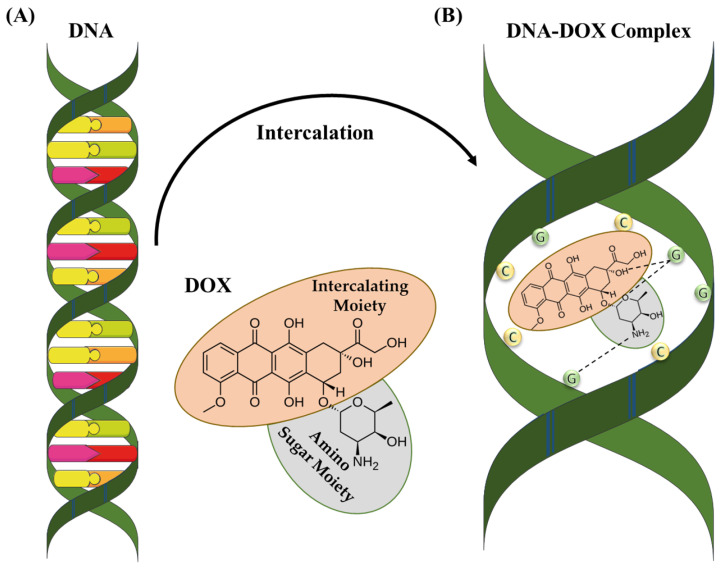
Schematic explanation of the chemical structure of DOX and its binding capability to DNA molecules, and the inhibition of the DNA synthesis. (**A**) The structure of DNA, including the intercalating DOX molecule. (**B**) The binding of DOX into double-stranded DNA molecules, adapted from Ref. [111].

DOX is a chemotherapeutic drug that has been approved by the FDA for cancer treatment [103,104,105]. It is the most commonly used therapeutic drug due to its ability to control the rapid cell division of tumor cells [106], and has been utilized in cancer treatment for its chemotherapeutic abilities. DOX has a molecular weight of 543 g/mol and is crystalline as a solid [107]. This molecule comprises a polar sugar moiety and nonpolar intercalating moiety. It exhibits maximum absorption at a wavelength from 489 nm to 500 nm [108]. DOX and its derivatives can form bonds with plasma proteins for transportation toward tissues. Generally, DOX can enter both the nucleus and cytoplasm of the cells. Once inside the cell, it has a higher affinity for DNA in the nucleus due to its mechanism of action involving DNA intercalation and topoisomerase inhibition [109]. DOX intercalates with DNA or RNA base pairs and inhibits them by blocking topoisomerase II (Figure 3) [110]. This enzyme plays a crucial role in cell proliferation. During DNA replication, topoisomerase II cuts off the filaments. After cutting the filament, DOX intercalates with the DNA molecule to disturb the mechanism. DOX is not restricted to a specific phase of the cell cycle but it exhibits maximum toxicity in the S phase of the cell cycle.

DOX can enhance cell death by various targets, including reactive oxygen species formation and senescence induction [112]. Reactive oxygen species (ROS) are produced in aerobic organisms through various processes, including the electron transport chain (ETC), catabolic oxidases, and peroxisome metabolism [113,114]. However, an excessive ROS production can cause DNA damage due to radicals acting on bases and the sugar–phosphate backbone of DNA [115,116]. Unrepaired damage may result in cell-cycle arrest, senescence, and apoptosis [117,118]. DOX binds directly to cardiolipin on the inner mitochondrial membrane, resulting in ROS production [119]. Elevated ROS levels cause considerable damage to the mitochondrial structure, eventually leading to cell apoptosis. DOX-induced intrinsic and extrinsic apoptosis in cardiac cells is mediated by mitochondria-derived ROS and calcium. This is accomplished by a nuclear factor of the activated T-lymphocyte (NFAT)-mediated upregulation of the FAS antigen ligand (FASL) and the downregulation of the FLICE/caspase-8 inhibitory protein (FLIP) [120]. In investigations assessing the influence of DOX on death ligands in induced pluripotent stem cell-derived cardiomyocytes, the upregulation of death receptors, such as tumor necrosis factor receptor 1 (TNFR1), FAS, and death receptor 5 (DR5), was observed. This upregulation intensified apoptosis, particularly in conjunction with the TNF-related apoptosis-inducing ligand (TRAIL) [104]. The substantial cardiotoxicity and thrombocytopenia associated with DOX significantly limit its therapeutic applications [121].

Similarly, it is commonly accepted that the application of chemotherapeutic drugs to cancer cells can induce senescence. Cellular senescence can also be induced in normal cells as a response to chemotherapy. This side effect can be minimized by using specific targeted therapy approaches. Consequently, therapy-induced senescence (TIS) has become an attractive approach for combating cancer with reduced side effects. However, recent findings suggest that cancer cell senescence could have adverse consequences, as senescent cells tend to create a procancerogenic environment. In addressing this challenge, scientists have introduced a novel pharmacological category called senolytics, specifically designed to eliminate senescent cells [122]. Senescent cells exhibit distinctive features, including cell-cycle arrest, the expression of senescence-associated β-galactosidase, the formation of heterochromatin foci, telomere shortening, the hypermethylation of histone H3K9, and the secretion of various factors, such as chemokines and inflammatory molecules, including MMPs, IL-1, IL-6, and IL-8. This secretory pattern is recognized as the senescence-associated secretory phenotype (SASP) [123]. Additionally, disrupted Ca^2+^ uptake has been demonstrated to instigate apoptotic processes and facilitate necrotic cell death. Notably, DOX has been identified for its ability to perturb mitochondrial and cellular Ca^2+^ homeostasis, making it a potential target for therapy [124]. However, the medical application of DOX is limited due to its high toxicity. Mostly drug transporter and antiapoptotic aspects are assumed as the main reasons for DOX resistance. For example, P-glycoprotein (a membrane protein) is a very popular drug transporter that creates DOX resistance through a lack of accumulation in tumor cells [125]. P-glycoprotein has two pseudosymmetric halves in the structure. Every half consists of a nucleotide-binding domain (NBD) responsible for the binding and hydrolyzation of ATP, as well as a transmembrane domain (TMD) [126]. Conformational changes arise in the structure of the P-glycoprotein when the drug binds with it, in which the drug is bound to one site and released to another site of the P-glycoprotein [125]. These sites of P-glycoprotein can be targeted by NPs to inhibit its activity [127].

## 6. DOX–Chitosan-Mediated NPs for Drug Deliveries

### 6.1. Active and Passive Drug Delivery

**Figure 4:**
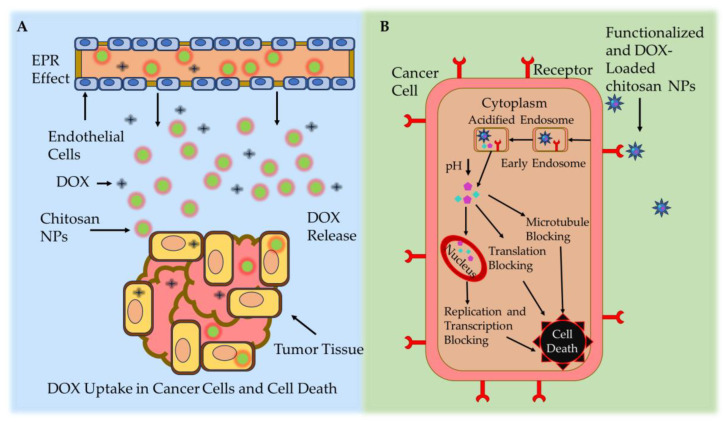
Chitosan-based NP drug delivery mechanisms. (**A**) Passive targeting mechanism: leaky tumor vessels release the DOX-loaded chitosan NPs at the cancer site via the EPR effect. (**B**) Active targeting mechanism: DOX-loaded chitosan NPs accumulate in cancerous cells via ligand-mediated endocytosis [139].

Effective drug delivery to tumor cells via chitosan NPs is divided into two categories (active and passive) (Figure 4). In passive DOX delivery, chitosan NPs accumulate in tumor tissue through leaky or defective vessels using the permeability and retention (EPR) effect [128]. NPs carrying anticancer drugs can easily navigate through the blood vessels in the angiogenic tumor site. This characteristic leads to a higher concentration of these NPs in tumor tissue compared to natural anticancer drugs, a phenomenon referred to as the EPR effect [129]. Once a solid tumor achieves a specific size, the surrounding normal vasculature becomes inadequate to meet the increasing oxygen demands for tumor development. Subsequently, as tumor cells undergo cell death, they release growth factors that stimulate the formation of new blood vessels from nearby capillaries [130]. Angiogenesis denotes the rapid formation of novel and irregular blood vessels, characterized by a disrupted epithelium and the absence of the basal membrane typically present in normal vascular systems. Due to the vascularization needed by rapidly growing tumors, coupled with restricted lymphatic drainage, the resulting irregular vascular architecture gives rise to an amplified EPR effect [131]. However, passive targeted drug delivery exhibited lower therapeutic efficacy and systemic side effects [132].

The targeted delivery of chemotherapeutic drugs presents dual benefits. Firstly, precise delivery to the targeted site reduces the requirement of the dosage, enhancing the efficacy of the therapy method [133]. Secondly, by reducing the overall drug dosage, the manifestation of drug-induced adverse effect is either prevented or significantly minimized. Nanomedicine therapy influences the diverse active and passive targeting capabilities of NPs to deliver drugs to specific target site [134]. Because of these potentials, NPs are used as viable methods for overcoming the drawbacks of traditional cancer therapies, such as nonselective toxicity and drug resistance. Tumor-targeted drug delivery takes use of the differences between malignant and healthy tissues [135]. During tumor progression, the tumor microenvironment changes. Inadequate oxygen supply and glucose to lactate conversion, caused by increased metabolism and growth rates, lead to a fall in the pH of the tumor tissue. This change, in conjunction with hypoxia and glucose deprivation, increases angiogenesis, a mechanism critical for tumor proliferation, migration, and maintenance [136,137]. Many tumors have an overexpression of certain antigens, including on their surfaces, making them potential drug delivery targets. This approach is successful as long as the selected targets for a specific cancer cell type can be recognized with confidence and are not expressed in considerable amounts elsewhere in the body [122,123]. An investigation elaborated on the synthesis of the active targeted water-soluble delivery of DOX [138]. This research involved two biodegradable and biocompatible biopolymers, poly-γ-glutamic acid (PGA) and chitosan. The self-conjugation of these two polymers produced stable and negatively charged NPs with an 80-150 nm diameter. The targeting agent was folic acid and bonded with the NP surface and polyanion. In this study, the stability of NPs, the toxic effect, the active targeting effect, and the DOX release efficiency was examined in in vivo situations. The results showed that the DOX-loaded NPs induced the DOX delivery compared to free drugs without damaging the normal cells. Table 2 shows the DOX deliveries via chitosan NPs with different combinations of NPs for cancer treatment.

### 6.2. Modified Chitosan–DOX Drug Deliveries

#### 6.2.1. Amino Acid-Modified Chitosan NPs

Amino acids are crucial molecules for all living cells; essential and nonessential amino acids play a key role in cell growth and proliferation. In the tumor microenvironment, the rapid growth and proliferation of the tumor cells require more amino acids to synthesize protein [163]. Traditional drug delivery approaches have focused on “starving cancer cells to death” by blocking nutrient intake [164]. Due to the hydrophilic nature of amino acids, they cannot cross animal cell membranes [165]. The transfer of amino acids into the cell requires a specific transporter on the animal cell membrane [166]. Several amino acid transporters have been discovered to cross the animal cell membrane, categorized according to substrate specificity and coupling ions [166]. Consequently, amino acid transporters have been considered an emerging goal for cancer chemotherapy. In cancer cells, the blockage of amino acid transporters is more specific and avoids unwanted nontarget effects [164,165]. On the other hand, chitosan NPs modified with amino acids can serve various purposes. Amino acids have the potential to improve the transport of therapeutic drugs, stabilize the nanoparticles, and allow specific interactions with biological targets. This specificity offers an opportunity for targeted tumor therapy, such as application in boron neutron capture therapy (BNCT), positron emission tomography (PET), and chemotherapeutic DDS [167,168]. Nowadays, therapy with amino acids (TAAI) for cancer treatment has gained more attention. A new method has been designed using amino acids and polymers for cancer treatment [169]. DOX-loaded chitosan glutamic acid (CS-Ga–DOX) NPs were prepared through the ionic gelation method. CS-Ga–DOX NPs exhibited a positive zeta potential and spherical structure. At a pH of 5.5 and 7.4, DOX was continuously released with a mutual burst. CS-Ga–DOX NPs have great potential as a pH-responsive nanocarrier for anticancer chemotherapy.

#### 6.2.2. Vitamin-Modified Chitosan NPs

Metastasis and stemness are the two main challenges in cancer treatment. Both have a solid connection with drug resistance and a low prognosis, finally leading to the failure of the cancer treatment. It has been described that cancer chemotherapy, particularly with the DOX in breast cancer treatment, can enhance metastasis and stemness. A combination therapy is an efficient method to suppress tumor cells with an enhanced synergistic effect. An advanced therapeutic system was developed by the combination of all-trans retinoic acid (ATRA) with DOX to resolve metastasis and stemness [170]. ATRA is a newly identified Pin1 (specific isomerase highly expressed within various tumor cells). It can effectively stop numerous cancerous pathways by successfully inhibiting and degrading Pin1. In this approach, researchers successfully synthesized a folic acid–chitosan-based polymer with both DOX (FA-CSOSA/DOX) and ATRA (FA-CSOSA/ATRA) NPs for cancer treatment. Due to the presence of FA, the uptake of these NPs was enhanced in cancer cells through folate receptors. This approach has a better synergistic effect as compared to DOX alone in the 4T1 cell line. In vivo, these NPs exhibited an 85.5% tumor inhibition, 2.5-fold higher than that of DOX–HCl alone. Another study was designed to synthesize vitamin E succinate–chitosan–histidine (VCH) multiprogram DOX carriers [171]. The π-π stacking bond between VCH and DOX was confirmed using a UV–vis spectrum. Drug release experimentations showed better pH sensitivity and sustained release results. DOX/VCH NPs were effectively taken up by HepG2 tumor cells, with the suppression rate reaching up to 56.27%. These NPs could combine with histidine and chitosan to attain pH sensitivity and P-gp inhibition, effectively suppressing the tumor cell growth and improving solubility. These multiprogram NPs show promise as an effective drug carrier in cancer chemotherapy without causing damage the healthy cells.

#### 6.2.3. Antibody-Modified Chitosan NPs

**Figure 5:**
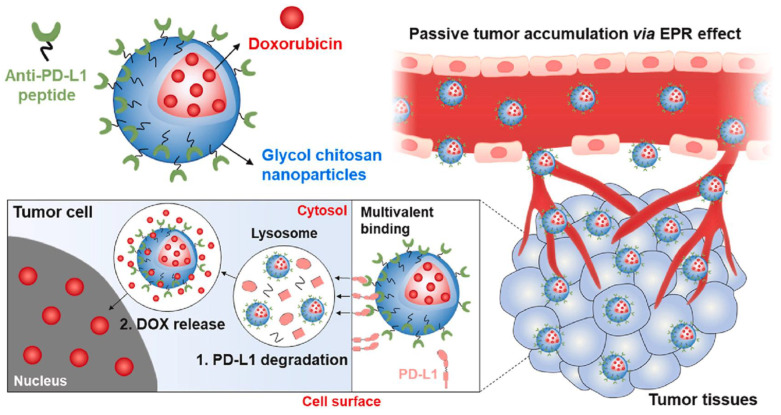
DOX–PP–CNP-based immunotherapy for ICD and PD-L1 degradation. All-in-one NPs, anti-PD-L1 peptide-conjugated, and DOX-loaded glycol chitosan NPs (DOX–PP–CNPs) were created, and both passive and active tumor targeting allowed the DOX–PP–CNPs to aggregate in targeted tumor cells. Then, the DOX–PP–CNPs improved the PD-L1 multivalent binding on the surface of tumor cells, which internalized to favor the PD-L1 intracellular trafficking to lysosomes as an alternative to recycling endosomes. Reprinted with permission from Ref. [146]. Copyright 2023, Elsevier.

Antibody–drug conjugates (ADCs) constitute a class of cancer cell-targeting drugs that have gained consideration. Monoclonal antibodies (mAbs) were synthesized to target antigens, and this approach was developed with the advent of hybridoma technology in 1975 [172]. Several mAbs have since gained approval, with herceptin being an example used in breast cancer treatment by targeting the HER2 receptor. However, mAbs alone are not enough for cancer treatment due to their low cytotoxicity in cancer cells [173]. Therefore, a new approach was designed to increase the cytotoxic effect in cancer cells. According to this strategy, mAbs were conjugated with biopolymers to enhance the drug efficiency. Similarly, chitosan NPs combined with two types of mAbs (anti-hMAM and anti-HER2) were synthesized for the treatment of breast cancer [141]. These PEGylated DOX-loaded CSNPs with mAbs exhibited enhanced cytotoxicity against MCF-7 cancer cells as compared to DOX-loaded CSNPs without mAbs. The synergetic therapy of immunogenic cell death (ICD) and the immune checkpoint blockade (ICB) has revealed extraordinary results against various types of cancers. For a safe and effective synergetic immunotherapy, researchers proposed all-in-one glycol chitosan NPs (CNPs) that administered anti-PD-L1 peptide (PP) and DOX to target tumor cells [146]. Briefly, a hydrophobic 5-cholanic acid was conjugated to the hydrophilic glycol chitosan backbones to create CNPs (Figure 5). Furthermore, the CNPs’ free amine groups were modified with tumor-targeting antibodies and peptides for targeted tumor drug delivery [174]. PP–CNPs thus avoided subcellular PD-L1 recycling, eventually abolishing the immunological escape mechanism in CT26 colon tumor. When the DOX–PP–CNPs were intravenously injected into mice with CT26 colon tumors, PP and DOX were successfully delivered to the tumor tissues via NP-derived passive and active targeting. This enhanced both lysosomal PD-L1 degradation and substantial ICD, ultimately leading to tumor regression through an antitumor immune response.

#### 6.2.4. Hyaluronic Acid-Modified Chitosan NPs

Hyaluronic acid (HA) is a polysaccharide found in the extracellular matrix of connective tissues. Structurally, HA has repeating units of N-acetyl-D-glucosamine and D-glucuronic acid connected with the β-1,3 and β-1,4 glycosidic linkage [175]. HA has been extensively used in actively targeted deliveries due to the high attraction for CD44 receptors (overexpressed in stem cells of cancer). The active targeted anticancer drug delivery with HA can enhance the solubility and effectiveness. In this regard, catechol (Cat)-modified chitosan@HA NPs were synthesized to deliver the DOX [176]. The Cat moiety enabled the carriers with good adherence and a constant local distribution of DOX. The ionic gelation method was used to prepare Cat-NPs from Cat-functionalized chitosan and HA. These prepared NPs have a negative charge and spherical shape. As compared to unmodified NPs, these NPs showed better mucoadhesive properties in oral mucosal tissues. In this method, DOX was loaded onto the modified NPs with a high loading capacity of 250 μg/mg, and sustained release was achieved. DOX-loaded Cat-NPs (DOX-NPs) inhibited the expansion of the HN22 carcinoma cell line. DOX-NPs were taken up, accumulated, and caused apoptosis in cells more rapidly as compared to free DOX. The findings showed that the prepared Cat-NPs have great potential and could be used as a novel carrier for the local delivery of DOX to oral cancer cells. Another study explained the HA-modified chitosan NPs for DOX delivery for effective cancer chemotherapy. Magnetic NPs containing chitosan/HA complexed with *κ*-carrageenan were prepared by using the solution method (hydrothermal method) [177]. An MTT assay was performed to check the effect of these magnetic NPs on MCF-7 and MDA-MB-237 cells. These MNPs have a spherical shape and a 100–150 nm diameter with a 74.1% DOX encapsulation capacity. However, the drug encapsulation capacity was enhanced by increasing the *κ*-carrageenan amount. Subsequently, the pH-stimulus-responsive drug was released in a sustained manner without side effects.

#### 6.2.5. PLGA- and PEG-Modified Chitosan NPs

PLGA and PEG are biocompatible and biodegradable synthetic polymers with the ability to increase stability and minimizing side effects. For example, pH-sensitive PEGylated chitosan NPs coated with PLGA were synthesized using the coassembly method to achieve effective DOX delivery for cancer chemotherapy [178]. The obtained DOX-loaded PEGylated chitosan/PLGA NPs (DOX–PCPNs) have a spherical structure, while the chitosan/PLGA formed a solid central core surrounded by hydrophilic PEG. DOX–PCPNs exhibited excellent stability and enhanced drug release in a serum-holding environment. Cytotoxic studies elaborated that the DOX–PCPNs were endocytosed, enhancing the DOX release in an in vitro environment and increasing DOX accumulation in cancer cells to improve antitumor efficiency. The hydrated shells of PEG protected against uptake by macrophage cells. In vivo results exhibited the ability of DOX–PCPNs to efficiently deliver DOX, reducing TRAMP-C1 tumor growth compared to free DOX. DOX–PCPNs exhibit great potential in drug delivery against tumor cells.

#### 6.2.6. Genetic-Material-Modified Chitosan NPs

**Figure 6:**
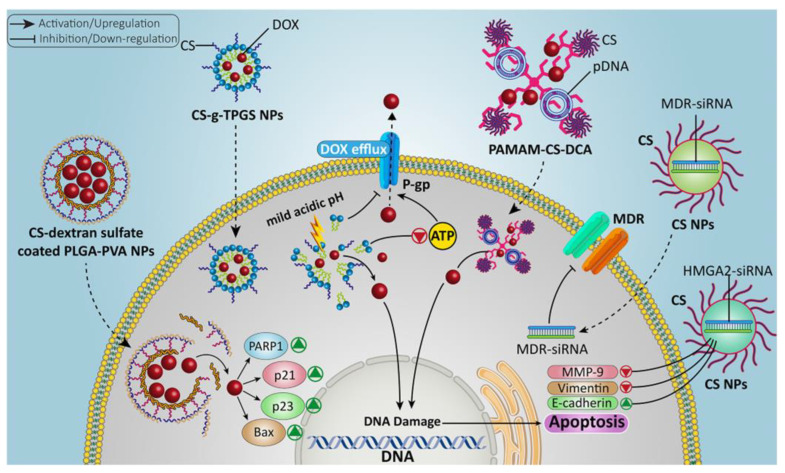
Various chitosan-based NP approaches for the DOX drug codelivery with genes leads to a modification in gene expression and cell apoptosis in cancer treatment. Reprinted from Ref. [201]. Copyright 2023, AIChE.

Sometimes, monotherapy against cancerous cells is insufficient for treatment. It has disadvantages, such as extreme toxicity in healthy cells and resistance [179,180,181]. Compared to conventional chemotherapy, gene therapy offers a safer route through nonviral vectors, but it exhibits limited efficiency and needs improvement [182,183]. Multidrug resistance (MDR) is also attributed to the malfunction of genes causing chromosomal changes in cancerous cells [184]. Many researchers have investigated gene delivery to treat cancerous cells with drug combinations [185]. The combined chemo- and gene therapy has provided an effective way for cancer treatment to overcome MDR due to its high synergetic effect [186,187,188]. Nucleic acids and genes require DDS because they are too large to enter the animal membrane. Moreover, gene therapy for cancer treatment is challenging due to the unstable and flexible nature of genes [179]. Here, we explored studies that highlight the use of chitosan-based NPs for the codelivery of DOX and genes in cancer treatment (Figure 6). Dendronized chitosan-based poly amidoamine deoxycholic acid (PAMAM-CS-DCA) NPs were synthesized [189,190,191]. These chitosan-based NPs exhibited low toxicity in healthy cells and a good gene transfection efficiency. Low doses of DOX increased the gene expression and synergetic ability, demonstrating great potential for the codelivery of genes and drugs. The overall results exhibited higher efficiency against tumor cells. Similarly, a double-walled microsphere was synthesized, and chitosan-based DNA nanocomplexes, including the p53 gene, were loaded for combined chemo- and gene therapy [192,193]. Another study described the chitosan-based NPs for the codelivery of DOX and siRNA. In this approach, carboxymethyl dextran (CMD) chitosan-based NPs were prepared to load the DOX and siRNA. The effect of these NPs on the epithelial–mesenchymal transition (EMT) gene expression and cell growth in HCT-116 cell lines was also explained [194]. In addition, another study elaborated the lung cancer therapy with HMGA2 (high-mobility group A2) suppressing small interfering RNA (siRNA) along with DOX by using chitosan-based NPs [195,196]. The findings showed that the codelivery of HMGA2, siRNA, and DOX through innovative CMDTMChiNPs was a novel therapeutic method with prodigious potential effectiveness for lung cancer treatment. The increased activity level and expression of P-glycoprotein were responsible for increasing the drug resistance [197,198]. To solve this, chitosan–g-D-α-tocopheryl polyethylene glycol (TPGS) NPs were produced by using evaporation techniques. TPGS NPs have the ability to control the P-glycoprotein activity and significantly decrease the ATP level, which is beneficial in preventing DOX efflux [199]. Chitosan–dextran–sulfate-coated PLGA–PVA (poly lactic-co-glycolic acid–polyvinyl alcohol) NPs for DOX distribution were used to test the effectiveness of the chitosan NPs by preventing DOX progression [200]. All the above-mentioned examples prove the effectiveness of codelivering DOX and genes using chitosan NPs as a carrier agent.

#### 6.2.7. Immunotherapeutic-Modified Chitosan NPs

Cancer leads to a significant proliferation of abnormal cells and immunosuppressive cells that inhibit the immune response [202,203,204]. Reforming the immune environment of tumors can enhance the efficiency of the immune response against tumors. [205,206,207]. Recently, tumor immunotherapy has gained a lot of attention for cancer treatment [208,209]. However, the efficiency and toxicity effects of simple immunotherapy are minimal [210,211]. Cancerous cells are present at the core tumor mass, and the treatment efficiency is restricted [212]. Due to this condition, combination therapy is the most effective method of cancer treatment [213,214]. A facile approach was developed using CMC derivatives (M2pep-CMCS) targeting tumor-linked macrophages 2 (TAM2) and cyclodextrin derivative (R6RGD-CM βCD) with the tumor target [215]. DOX was loaded onto cyclodextrin-derivative NPs, and R848 was loaded onto CMC-derivative NPs, demonstrating good absorption. These NPs enormously increased the expression levels of cleaved Caspase 3, indicating enhanced cell apoptosis. Similarly, these NPs also altered the shape of tumor-linked macrophages. Overall, these materials are considered an effective delivery carrier in cancer treatment. Another study has investigated enhancing the immune response against cancer treatment, in which TMC-based NPs were synthesized and fabricated with DOX and interleukin-2 (rhIL-2) [216]. These NPs proficiently delivered the hydrophobic DOX and hydrophilic interleukin-2 to attain the combination therapy against the tumor microenvironment. DOX was attached to the TMC NPs with covalent bonds that led to the pH-sensitive release, while interleukin-2 bonded through electrostatic forces. The diameter of these NPs was about 200 nm, and a zeta potential > 20 mV was recorded. For the targeted delivery, folate modification was used to accumulate the drugs at the target site. The final results showed that the combinational therapy of interleukin-2 and DOX-based TMC NPs have the potential to kill the tumor cells and enhanced the immune response against cancer.

### 6.3. Combined Delivery with Other Anticancer Drugs

A large number of studies have focused on combining chemotherapeutic drugs and chemosensitizers due to the resistance of tumor cells to DOX alone. The combined effect of anticancer drugs may improve the tumor suppression efficiency and minimize side effects [217,218,219,220]. Overall, the codelivery of drugs with chitosan NPs has proven the efficiency of this method against various cancerous treatments by reducing the drug resistance without harming the noncancerous cells [221]. In this aspect, research was conducted on breast cancer treatment. The codelivery of DOX and Tanshinone IIA (TSIIA) (a bioactive compound isolated from Chinese herbs known as “Danshen”) was achieved using CMC chitosan-based hypoxia NPs for breast cancer treatment [222]. Hypoxia-responsive NPs are designed to respond to low-oxygen levels, a condition known as hypoxia, typically found in tumor microenvironments. These NPs showed an improvement in release efficiency and an increase in the cytotoxicity of DOX against the tumor microenvironment. The immunofluorescence staining of the tumor section confirmed that the combined nanoparticles exerted a synergistic antitumor effect by inhibiting tumor. Consequently, CMC chitosan-based NPs exhibited promising properties for drug delivery against breast cancer. Degradation of the delivery agent is crucial for DDS at specific target sites and to prevent the presence of the delivery agent’s byproduct in the body [223]. Cancerous cells produce glutathione (GSH) 7 to 10 times more than healthy cells, which is highly beneficial for disulfide bond degradation [224,225]. Thus, a disulfide-bond-based DDS proves to be very helpful for enhancing the stability of delivery agents and facilitating degradation at the targeted tumor site in cancer therapy [226,227,228]. To achieve this purpose, another approach was established, wherein redox-sensitive chitosan/stearic acid NPs (CSSA NPs) were synthesized for DOX and curcumin delivery in cancer treatment [229]. The degradable CSSA NPs had a size of about 200 nm and were synthesized based on disulfide cross-linking. The hydrophilic DOX and hydrophobic curcumin drugs were encapsulated onto the CSSA NPs. Codelivery of therapeutic drugs through this approach increased the efficiency of the cancer treatment. These NPs exhibited a low drug release in the normal cells, while approximately 98% of DOX and 96% of curcumin were released in the tumor cells under a GSH reducing environment. Consequently, this method has demonstrated enhanced encapsulation, release of dual drugs, and cytotoxicity for cancer treatment. Another method was established for CD44 receptor targeting, reducing multidrug resistance (MDR) and enhancing the drug release and cytotoxicity in tumor cells for breast cancer treatment [230]. The NPs consisted of three layers: a poly core, liposome, and chitosan, respectively. These NPs (Ch-MLNPs) were loaded with DOX, silybin, and paclitaxel. These three drugs were released at target-specific sites exploiting CD44 receptors in breast cancer cells. In vivo, studies showed the good efficiency of these three-layered NPs against breast cancer and lowered the MDR.

## 7. Stimuli-Sensitive Deliveries of Chitosan–DOX NPs

### 7.1. Endogenous Stimuli-Sensitive Drug Deliveries

#### 7.1.1. pH-Sensitive Drug Deliveries

The cancerous cells have a pH of 6.5 (slightly acidic), which is lower than the physiological environment; drug release can be reduced due to that difference. The extracellular acidic environment is one of the most significant properties that differentiate healthy cells from cancerous cells [231]. For rapid progression, cancer cells choose aerobic glycolysis to continue the metabolic process, increasing the production of lactic acid and the accumulation of protons in the cell [231,232]. Nanocarrier derivatives can deliver the drug at a mildly acidic pH. The bond between the NPs and drug degrades (depending on the nature of bonds) in the mildly acidic pH of cancerous cells to release the drug at the target site [233]. Different bonds, like acetals, amine, oxime, ester, and amide, can be formed between the NPs and drugs [234]. The overcoming of the MDR, the effective release, and the accumulation of drugs are the necessary points to achieve. For this purpose, dual-pH-responsive chitosan NPs were invented to overcome the MDR tumor (MCF-7/ARD) in human breast cancer [235]. In this study, chitosan NPs were sensitized to extracellular tumors with a pH of 6.5, which participated in the surface charge reversal through the breakage of β-carboxylic amide, enhancing the cellular uptake efficiency. Furthermore, these chitosan-based NPs also exhibited responsiveness to an intracellular pH of 5.0 in the tumor microenvironment, with poor blood perfusion and limited oxygen supply. This pH fluctuation played a crucial role in inducing the protonation of the amino group within the acidic environment [236,237]. Cells assays verified that dual-pH-sensitive particles caused induced toxicity in the MDR tumor cells. Furthermore, the NPs could overcome tumor resistance by decreasing the intracellular levels of ATP and PARP-1, ultimately receiving a stronger antitumor efficiency. Moreover, the amphiphilic chitosan NPs showed a high efficiency against cancerous cells. Further research explored chitosan/polyvinylpyrrolidone/hematite (CS/PVP/α-Fe_2_O_3_) nanocarriers for the drug delivery of DOX. The NPs had a spherical shape and could load Fe_2_O_3_ on CS/PVP. The CS/PVP-based NPs provided pH-controlled drug delivery for tumor treatment and suppressed the breast cancer cells [238].

**Figure 7:**
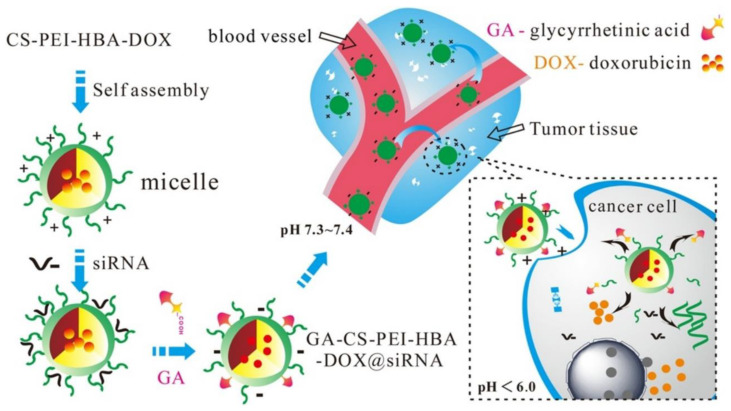
The controlled release of acid-sensitive micelles (GA-CS-PEI-HBA-DOX@siRNA) were produced. These micelles are excellent in delivering both DOX and siRNA to the tumor location, laying the groundwork for effective combined therapy. Adopted with permission from Ref. [241]. Copyright 2023, Elsevier.

Nanocarriers with pH-sensitive capabilities are used for chemotherapy and drug delivery. Due to the nonspecificity of DDS, it affects normal cells as well. In a research study, hollow mesoporous silica NPs (HMSNGM-CS-FA) were produced for the combined delivery of DOX and pheophorbide (PA) [239]. These NPs exhibited efficient drug release capabilities based on the pH-sensitive swelling effect of the coating layer. This study demonstrated significant encapsulation and drug release capabilities, resulting in enhanced cytotoxicity in cancerous cells based on pH-sensitive NPs. Another feasible method was adopted to produce the pH-sensitive surface-charge reversal CMC-based DOX DDS using an organic solvent-free coprecipitation approach [240]. According to this approach, DOX was loaded in the core of PDPA fragments. These fragments were combined with the PEGylated CMC to form a shielding shell. Overall, results demonstrated the good drug-loading and releasing capacity, leading to tumor cells death. In addition, a chitosan-based polymeric drug was prepared for the DOX drug codelivery with siRNA for the tumor cell treatment. These polymeric nanocarriers were entered through hepatoma cells exploiting glycyrrhetinic acid-receptor-mediated endocytosis. The DOX releasing concentration was 90.2% out of the total drug-encapsulated concentration, and the siRNA releasing concentration was 81.3% (out of 50 μL of 100 μg/mL) after 10 h. This drug could suppress tumor cells by 88% (out of the total tumor size) through chemonucleic acid therapy (Figure 7) [241]. A high pH usually decreases the solubility of chitosan. Similarly, polyvinylpyrrolidone (PVP) was used, but alone it reduced the drug release. To control these problems, the conjugation of chitosan and PVP has been recommended to enhance the solubility of chitosan at a high pH level [238,242]. Another method was used to successfully fabricate iron (III) carboxylate metal–organic framework NPs coated with a glycyrrhetinic acid–chitosan conjugate (MIL-101/GA-CS), which behaved as a pH-responsive and target-specific agent to deliver DOX for hepatocellular carcinoma (HCC) therapy (Figure 7) [243]. These NPs have the advantages of a uniform size, drug encapsulation effectiveness, and pH-dependent targeted drug release. In vitro cytotoxic effects revealed that the NPs had excellent inhibitory effects on HepG2 cells due to the continual release of DOX, although these NPs had no significant toxicity in normal cells. As a result, MIL-101-DOX/GA-CS NPs have the potential to be used in therapy as a pH-responsive controlled DDS.

#### 7.1.2. Redox-Sensitive Drug Deliveries

The redox imbalance represents another exclusive parameter within the tumor microenvironment that is accountable for increasing the rate of cancer proliferation [244]. The synthesis of ROS started by cancer cells, cancer-associated fibroblasts, and endothelial cells contributes to the progression of cancer cells. Further, glutathione (GSH) is an antioxidant and acts as a reducing agent for ROS and oxidative stress [245]. The combination of suppressor agents and chitosan has been used to produce nanocomposites for drug delivery improvement. Chitosan with oligosaccharide (CSO) and stearic acid (SA) can be utilized to synthesize glycolipids, specifically copolymers, and these demonstrate good capability in delivering drugs to the tumor microenvironment [246]. However, micelles synthesized from CSO–SA face a significant challenge of efficient drug delivery in vitro due to the slow degradation kinetics of the amide bond. To solve this problem, DOX could be coupled with the CSO–SA through the help of a disulfide bond. This method was beneficial in the production of CSO–SA-based nanocomposites (due to the high GSH level, the NPs were redox-sensitive) and could deliver DOX to eliminate the progression of breast cancer [247]. Another study has explored the redox-responsive chitosan NPs for DOX delivery. In this method, N, N′carbonyldiimidazole (CDI) catalysis generated amphiphilic low-molecular-weight chitosan–lipoic acid (LC-LA) conjugates with varying degrees of substitution (DS) of LA, which self-assembled into redox-sensitive micelles [248]. The diameter, zeta potential, biocompatibility, critical micelle concentration, and the redox-sensitive response of blank micelles were explored. According to the results, blank micelles with a low critical micelle concentration, nanosize, and positive zeta potential demonstrated excellent biocompatibility and redox-sensitive response. The DOX drug was loaded on these micelles for cancer treatment. The loading capability, drug-released behavior, antitumor efficiency, and cellular drug uptake were elaborated, indicating that DOX-loaded micelles have a good loading capability and show a redox-trigger response and solid antitumor efficiency against A549 cells. An increase in the DS of LA decreased the critical micelle concentration and cumulative release concentration of DOX while increasing the loading efficiency, antitumor capability, and cellular uptake of DOX-loaded micelles, which was caused by the increased contact of hydrophobic groups in the micelles with the DS of LA. In general, self-assembled LC-LA micelles with good biosecurity and redox-sensitive responsiveness capture favorable application diagnostics in DOX administration and provide an understanding on DOX’s cancer therapeutic impact.

#### 7.1.3. Enzyme-Sensitive Drug Deliveries

Enzymes, such as protease, phospholipase, or glycosidase, play a crucial role in biological mechanisms, and abnormalities in the enzyme function can lead to a disorder like cancer [249]. Enzyme-dependent NPs have gained incredible consideration due to target specificity towards cells that overexpress enzymes [212]. The encapsulated drug in NPs is released at the target site due to the specific enzyme function [250]. Enzyme-responsive moieties are attached covalently or noncovalently with the polymers to obtain the desired NPs. However, covalently bonded NPs may disturb the function and target specificity of the enzymes due to suboptimal reactivity with the NPs [250]. On the other hand, noncovalently bonded NPs are highly specific to the target. A few studies have explained the enzyme-sensitive DOX delivery via chitosan NPs against a tumor environment. For example, hollow mesoporous silica spheres (HMSSs) were synthesized and linked with chitosan by the azo linkage (HMSS-N=N-CS) for enzyme–stimulus colon-specific drug delivery [251]. After that, DOX was loaded into the pores of HMSS-N=N-CS. These NPs showed stability, biocompatibility enhancement, and reduced protein adsorption on HMSSs. The final results revealed an increase in the cellular uptake of the drug after enzyme incubation.

### 7.2. Exogenous-Stimuli-Sensitive Drug Deliveries

#### 7.2.1. Light/Photo-Sensitive Drug Deliveries

Photosensitizers have found applications in stimuli-sensitive systems, involving the chemical modification of chitosan to respond to stimuli, such as UV and light, in photodynamic therapy (PDT) for treating cancer cells [252]. Several methodologies have been proposed for utilizing chitosan-based NPs in light-dependent delivery. Light-sensitive NPs, characterized by a sphere-shaped structure with functional-group-rich surfaces exhibiting the enhanced EPR effect, were modified with polymers like poly (D, L-lactic-co-glycolic acid) PLGA and poly (ε-caprolactone) PCL. This modification enhances the biodegradability and biocompatibility of the NPs, allowing for intravenous injection [253]. These NPs exhibited a good encapsulation efficiency and cytotoxicity in the tumor microenvironment [254]. In another experiment, a light-dependent platform was developed consisting of DOX-loaded gold NPs for breast cancer. In addition to photothermal therapy, it was observed that DOX produced oxidative stress via ROS [255]. Another study was performed in which the photosensitizer chlorin e6 (Ce6) with DOX-encapsulated chitosan (CS)–tripolyphosphate (TPP) NPs were synthesized for cancer therapy [160]. The ionotropic gelation method was used to prepare these NPs, and their photophysical and morphological properties were studied. The Ce6 was loaded onto the NPs through the self-assembly of chitosan with TPP–DOX under an aqueous environment. The prepared NPs had an 80–120 nm diameter with a negative zeta potential of -6 mV. The absorption spectrum of Ce6-coated NPs was the same as free Ce6, suggesting that the Ce6 chromophore underwent no alterations as a result of the coating. These NPs exhibited strong photostability and singlet oxygen generation (SOG). The Ce6-fabricated and DOX-encapsulated NP size was about 90–130 nm, and the charge was about −30 mV. The results exhibited high-DOX-encapsulation capacity and pH-controlled release. Moreover, these NPs exhibited a high uptake of DOX drug under irradiation at near-infrared (NIR) ranges against MCF-7 cancerous cells. This study showed how NPs could be used to release DOX under photocontrol in a tumor microenvironment.

#### 7.2.2. Magnetic-Sensitive Drug Deliveries

Magnetic-sensitive NPs can be synthesized using various methods, and their properties are often tailored for specific applications. One common approach involves incorporating magnetic materials into the nanoparticle structure. This technique relies on the magneto-sensitive moiety in the presence of a strong external magnetic field [2]. Magnetic flux can be changed for guiding the nanocarrier to deliver the drug to a specific target site. Several superparamagnetic iron oxide NPs (SPION) are prepared with moieties that can specifically bind at the desired position. The interaction between the external magnetic field and SPION raises the temperature at the tumor site, called the “magnetic thermal ablation” [2]. A gradient magnetic field can enhance the uptake of NPs into tumor cells and deliver the maximum drug at the target site. Chitosan-based magneto-sensitive NPs are capable of crossing the blood–brain barrier. Mostly, chitosan-containing magnetic NPs (MNPs) have a magnetic nucleus inside and a biodegradable outer shell. The inner magnetic core is responsible for transporting the NPs to the specific target site, and the outer biodegradable shell releases the drug. MNPs are highly active and readily oxidized in air, leading to a loss of magnetism. Chitosan preserves these MNPs from oxidation and reduces their toxicity. Chitosan also facilitates the binding of MNPs with various functional groups of the DOX drug moiety, resulting in less aggregation, a longer half-life, and enhanced stability. Fe_3_O_4_, ZnFe_2_O_4_, CoFe_2_O_4_, Fe_2_O_3_, and other magnetic particles are utilized in the synthesis of chitosan-based NPs. In this regard, recent research elaborated on the magnetic-sensitive chitosan-NP-based DOX delivery for cancer therapy. According to this study, pH-sensitive NPs were produced using chitosan and succinic anhydride (CSSA) for the targeted delivery of DOX to osteosarcoma cells [155]. First, they synthesized CS–folic acid (FA) conjugates by the amide linkage of chitosan with FA. Following that, CS-SA/CS-FA was produced by coating of Fe_3_O_4_ (MNPs) ferrofluid. Then, DOX molecules were placed onto the CS-SA/CS-FA NPs. The DOX release profiles at various pHs revealed that the DOX release was boosted in acidic environments. The MG-63 cells, which express folate receptors, demonstrated much better cellular absorption of the DOX-loaded CS-FA/CS-SA@MNPs than the lung cancer A549 cells. The cytotoxicity experiment revealed that these NPs exhibit cytocompatibility with MG-63 cells.

#### 7.2.3. Ultrasound-Sensitive Drug Deliveries

Ultrasound-responsive NPs synthesized with chitosan have been extensively examined for their biosafety towards healthy cells. Ultrasound-triggered drug delivery has gained consideration as a noninvasive modality against cancerous cells. A study supports this noninvasive modality, in which a hydrophobic DOX drug was encapsulated onto the palmitoyl-modified glycol chitosan amphiphile (PmGCA) NPs [256]. The reappearance of the DOX fluorescence peak after 2 MHz under ultrasound exposure proved the drug release. In vivo, the results exhibited remarkably lower fluorescence in the liver and heart as compared to DOX alone. This approach is considered beneficial for DOX drug delivery for cancer treatment, with low side effects on the major organs. Similarly, other chitosan-derivative-based NPs were synthesized for the DOX delivery for efficient cancer treatment. O-carboxymethyl chitosan/perfluorohexane nanodroplets (O-CS NDs) encapsulated with DOX were tested in vitro [257]. O-CS NDs attained higher tumor cellular penetrations at an acidic pH, a good ultrasound imaging capability, and strong cytotoxicity. This research explained the improved cell interaction ability under ultrasound exposure and targeted DOX delivery against cancer cells.

### 7.3. Multisensitive Drug Deliveries

**Figure 8:**
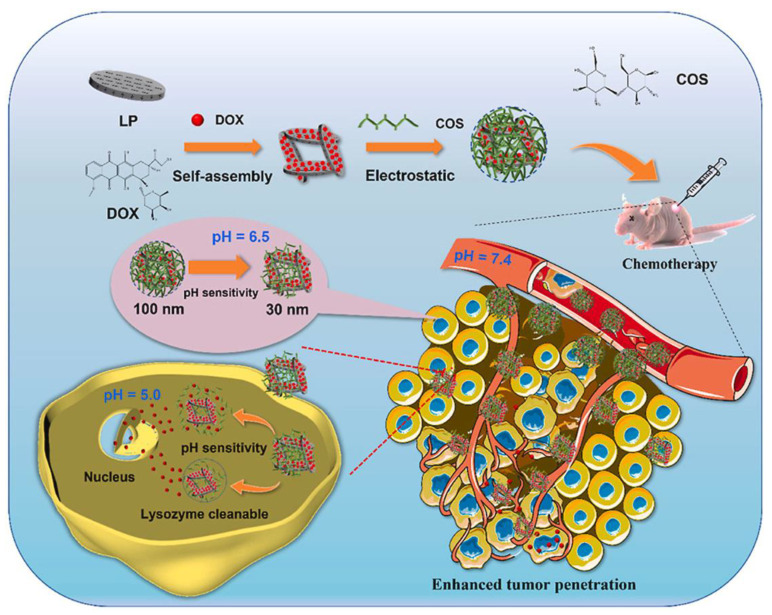
Schematic presentation of the production of LDC (a compound of laponite (LP), doxorubicin (DOX), and chito-oligosaccharides (COS)) NPs and their anticancer activity. Due to the presence of enzymes in the tumor microenvironment, LDC NP sizes become smaller, from 100 nm to 30 nm. After cellular uptake, the enzymatic and acidic pH environment enhances the DOX release. Reprinted with permission from Ref. [262]. Copyright 2023, Elsevier.

Multistimuli-sensitive systems are designed to respond to a combination of stimuli, providing a more sophisticated and controlled drug release profile. Such systems are often explored to enhance drug delivery precision and efficiency, especially in targeted therapy. [258,259,260]. In this regard, chitosan NPs with folate-covered dual-responsive mesoporous silica NPs (MSNs) actively targeted the tumor cells and provided efficient drug delivery [261]. These NPs were produced with a very economically cheap silica and sodium silicate source. When the DOX was loaded into the MSNs, it reacted with cystamine dihydrochloride, and then a folate conjugate was created to produce dual-stimulus-responsive NPs. DOX was released at the target from the MSNs under an acidic pH (a pH of 5.5, 10 mM GSH) and redox environment in vitro. These NPs showed 2.14-times-enhanced cytotoxicity in MCF-7 cells and 1.65-times-enhanced cytotoxicity in MDA-MB-231 cells as compared to DOX alone. Low stability, unpredictable drug release, and limited tumor penetration hinder the applicability of nanomaterial-based DDS. To solve this, pH- and enzyme-responsive shrinkable NPs were created [262]. LDC (a compound of laponite (LP), doxorubicin (DOX), and chito-oligosaccharides (COS)) was the main component of these double-responsive NPs. LDC NPs have a diameter of 100 nm, and DOX drugs can accumulate effectively in the tumor ecosystem under an in vivo environment. Lysozymes, found in the extracellular environment, facilitated the degradation of chitosan oligosaccharides (COSs) and resulted in the formation of smaller DOX-based nanoparticles. Additionally, they induced a size reduction in LDC nanoparticles, reducing their original size from 100 nm to 30 nm. This size reduction enhances the drug’s ability to penetrate deeply into tumor cells, as illustrated in Figure 8. Additionally, after entering the tumor tissues, the acidic and enzymatic cellular environment triggered a rapid release of DOX, leading to the quick death of cancer cells. LDC NPs often exhibited no significant cytotoxicity in the mice’s primary organs. The outcomes of this research exhibited that these LDC NPs possess efficient targeting capabilities for tumor tissues with controlled drug release. Another study supported the use of multiresponsive chitosan-based NPs for cancer therapy. In this method, triple-responsive (pH, redox, and ultrasound) hybrid NPs were produced for controlled and sustained drug delivery. These hybrid NPs were manufactured through a mesoporous-silica-coated magnetic core (Fe_3_O_4_@SiO_2_@mSiO_2_) and pH/redox-responsive polymer layer [263]. The pH/redox-responsive polymer (CS-LA) layer of the particles was created using chitosan (CS) and lipoic acid (LA). Then, Fe_3_O_4_@SiO_2_@mSiO_2_ NPs were fabricated using the CS-LA polymer. The results showed that the hybrid NPs efficiently delivered DOX under cellular pH differences. Additionally, the results of the dual pH/redox-responsive drug delivery demonstrated that the effective and controlled drug release profile was shown by the hybrid NPs at a pH of 5.5 in the MCF-7 cells. These hybrid triple-triggered NPs are well-suited for prolonged and regulated drug delivery in vivo.

## 8. Conclusions and Future Perspective

In comparison to traditional treatment methods, nanotechnology has emerged as a highly promising opportunity for cancer chemotherapy. To specifically target tumors, it is necessary to choose suitable and efficient targeting carriers [264]. Chitosan is the second most abundant natural polysaccharide, has recently gained more consideration, and extensive research has been conducted to demonstrate its drug delivery abilities and NP synthesis efficiencies [265,266,267]. Chitosan can be modified with different molecules for various purposes and requirements in drug delivery for cancer treatments [69,70]. Preclinical and clinical experiments have been conducted on chitosan due to its biocompatibility and cytotoxicity in cancerous cells. On the other hand, DOX is the most extensively used drug for cancer treatment. Although DOX has exhibited a toxic effect in the tumor environment, it has also damaged normal cells due to its nonspecificity. Moreover, DOX has also faced some resistance. To solve this problem, scientists have used chitosan NPs to overcome drug resistance and enhance the specificity of tumor cells without damaging healthy cells [140,144]. This review mainly focused on the importance of chitosan NPs and DOX deliveries using these NPs for cancer treatments. Chitosan NPs can be prepared and modified using different convenient methods. We have also discussed the basic mechanism of action of DOX and intercalation to DNA for tumor elimination. Chitosan NPs can be combined with other agents, enhancing their efficiency in drug delivery. Cancerous cells exhibit an acidic pH slightly lower than the normal physiological pH. Scientists have developed pH-sensitive chitosan NPs to deliver the DOX based on the pH difference [243,268,269,270]. Similarly, different stimuli-sensitive chitosan NPs have been developed, including redox-sensitive [248,271,272,273,274], enzyme-sensitive [275], light-sensitive [276], magnetic-sensitive [155,277], ultrasound-sensitive [256,278], and multistimuli-sensitive [261,262] chitosan NPs, to deliver DOX at target sites according to specific needs. These stimulus-responsive NPs have enhanced the DOX release at specific target sites. Chitosan possesses anticancer capabilities and produces a synergistic influence with DOX. Moreover, chitosan-based NPs also improve the internalization of DOX into tumor cells and help to increase the cytotoxicity in the tumor microenvironment. It also reduces the DOX resistance as well as the MDR.

Chitosan can be easily modified, making it a more valuable drug delivery agent for releasing drugs at the target site according to environmental needs. Lots of research exists on chitosan-based NPs for cancer treatments; further experiments can be applied in clinical trials. If chitosan NPs are used in clinical trials, they will become more efficient over time. Although chitosan NPs have good biocompatibility, they can still cause damage to some healthy cells. There is still a need to develop approaches for treating cancer patients with the complete benefits of chitosan NPs in clinical trials. DOX has severe toxicity in both cancerous and healthy cells. Chitosan NPs reduce the toxicity of DOX through target-specific delivery. However, we still need to develop unique methods to minimize the cytotoxic effects in normal cells. This goal can be achieved through combination therapy of DOX with other therapeutic molecules, immunotherapeutic agents, and genes to make it target-specific and achieve better synergetic effects with minimal damage to healthy cells.

## Figures and Tables

**Table 1:** Different derivatives and modifications of chitosan, their functional groups, properties and applications.

Derivatives/Modifications	Specific Properties	Biomedical Applications	Ref.
Chitosan	Biocompatibility; Biodegradability; Mucoadhesive	Drug delivery; Gene delivery; Wound healing	[42,43,44,45]
Trimethyl chitosan (TMC)	Soluble in alkaline solution; Positive charged	Mucoadhesion drug delivery; Antibacterial applications	[69,70]
PEG-conjugated chitosan	Improved solubility; Stability	Chemotherapeutics delivery; enhanced encapsulation efficiency	[86,87,88]
Carboxymethyl chitosan (CMC)	Increased solubility; pH sensitivity; Paracellular permeability	Medically hemostatic; Conjugation with antibodies	[74,75,89]
Thiolated chitosan (TC)	Stability; Permeability	Wound healing; Effective drug release	[78,79,80,81]
Quaternary ammonium derivatives	Water soluble; Positively charged; Permeability; Mucoadhesion	Therapeutic drug carrier; Targeted delivery	[72,73]
CS–drug conjugates	Sensitivity; Stability; Prolonged circulation time	Tumor site target; Macromolecule release at target site	[90,91]
Poly butyl acrylate chitosan	Thermal stability; Structural integrity	Drug delivery carrier	[92,93]
Glycated chitosan (GC)	Solubility; Nontoxic; Hydrophilic	Coating; Catalyst; Drug releasing	[84,85]
Hyaluronic acid-conjugated chitosan	Increased stability in vivo; Prolonged circulation time	Enhance the antitumor ability; Increase drug accumulation in tumor cells	[94,95]
Folic acid-conjugated chitosan	Stability; High affinity for folate receptor	Drug uptake; Drug accumulation in colorectal cancer	[96,97]

**Table 2:** Active and passive targeted drug deliveries by using chitosan NPs for DOX drug delivery in cancer treatments.

NP Types	Cancer Type or Cell Line	Drugs	Active or Passive	Remarks	Ref.
Chitosan and O-HTCC (ammonium-quaternary derivative of chitosan) NPs	Kidney and osteosarcoma cancer/Vero and SaOs-2 cell lines	DOX	Passive	High encapsulation but low releasing capacity.	[140]
PEGylated chitosan NPs	Breast cancer/MCF-7 cell line	DOX	Active	Three-times enhanced cytotoxicity.	[141]
DSe-CMC (diselenide-cross-linked carboxymethylchitosan) NPs	Liver cancer/HepG2 and H22 cell lines	DOX	Passive	Releasing capacity enhanced below the acid and redox environment.	[142]
L61-OE-CS (acid-labile ortho-ester-modified pluronic and chitosan) NPs	Liver/HepG2 and H22 cell lines	DOX	Passive	Drug releasing rate was enhanced at an acidic pH.	[143]
Chitosan/alginate NPs	Breast cancer/MCF-7 and MDA-MB-231 cell lines	DOX and HCQ	Passive	Inhibited the autophagic degradation and enhanced the drug delivery.	[144]
Chitosan–MgFe_2_O_4_ magnetic NPs	Breast cancer SKBR-3 cell line	DOX	Passive	An 84.28% encapsulation efficiency and an 85.86% releasing capacity.	[145]
PP-CS (anti-PD-L1 peptide and chitosan) NPs	Colon cancer/CT26 cell line	DOX	Active	Strong synergetic immunogenic response and induced tumor regression.	[146]
CS-PAPBA (chitosan–poly(*N*-3-acrylamidophenylboronic acid) NPs	Liver cancer/H22 cell line	DOX	Active	Enhanced the deep penetration and accumulation in tumor cells.	[147]
LGCC (lactobionic acid–guanidinobenzoic acid–cystamine bismethacrylamide-cross-linked chitosan-poly(methyl methacrylate))NPs	Breast cancer/CXCR 4 cell line	DOX	Active	Significant suppression of CXCR 4-positive hepatocarcinoma and breast cancer cells.	[148]
Chitosan NPs/CMD (carboxymethyl dextran)	Lung cancerA549 cell line	DOX and IGF-1R siRNA	Active	Synergistic result of DOX cytotoxicity and apoptosis in cancerous cells.	[149]
Chitosan–SPIO (superparamagnetic iron oxide) magnetic NPs	Ovarian cancer/A2780 and OVCAR-3 cell lines	DOX	Passive	High-tumor-growth inhibition after 96 h of exposure.	[150]
HA (hyaluronic acid)-chitosan NPs	Breast cancer/MDA-MB 231 cell line	DOX–miR-34a	Active	Codelivery enhanced the efficiency and reduced the resistance and side effects.	[151]
Chitosan–Raloxifene NPs	Breast cancer/MCF-7 cell line	DOX	Active	A 95% encapsulated and 60% DOX-release capacity; inhibited cell growth.	[152]
Chitosan NPs	Colorectal cancer/HT-29 cell line	DOX and HMGA2–siRNA	Passive	Combination was effective against tumor cells.	[153]
Modified chitosan NPs	MCF-7 and Caco-II cell line	DOX	Passive	Higher loading ability and effectively eliminated tumors.	[154]
CS-FA (chitosan–folic acid)/CS-SA-MNPs (succinic anhydride magnetic nanoparticles)	Lung cancer/MG-63 and A549 cell lines	DOX	Active	NPs deliberated as an effective pH-dependent nano-DDS.	[155]
FA (folic acid)–chitosan NPs	Liver cancer/HepG2 cell line	DOX	Active	Inhibited the cell cycle at the G2/M phase.	[156]
COOH–chitosan MSNs (mesoporous silica nanoparticle)	Breast cancer/TNBC and HER2 cell lines	DOX	Active	Enhanced drug release; increased the efficiency of DDS.	[157]
AAP-CS-NPs (Auricularia auricular polysaccharide–chitosan NPs)	Breast cancer/MCF-7	DOX –HCl	Passive	Enhanced cellular uptake compared to free DOX.	[158]
Chitosan–TPP NPs	Lung cancer/A549 cell line	DOX	Passive	Encapsulation efficiency of approximately 95% and a good cytotoxic effect.	[159]
Ce6–chitosan–TPP NPs	Breast cancer/MCF-7 cell line	DOX	Passive	Significant enhancement observed in the drug release rate.	[160]
Chitosan magnetic NPs	Breast cancer/MCF-7 cell line	DOX	Passive	Higher drug released at a pH of 4.2 as compared to a pH of 5.	[161]
CPCN NPs (collagen peptide chitosan nanoparticles)	Cervical cancer/HeLa cell line	DOX–HCl	Passive	Enhanced drug release and increased apoptotic cell rate.	[162]

## Data Availability

Not applicable.
